# Gut dysbiosis in neurodevelopmental disorders: linking microbiota signatures to cognitive rigidity in autism spectrum disorder

**DOI:** 10.3389/fmicb.2026.1760635

**Published:** 2026-01-27

**Authors:** Bhagavathi Sundaram Sivamaruthi, Periyanaina Kesika, Chaiyavat Chaiyasut, Durairaj Ragu Varman

**Affiliations:** 1 Office of Research Administration, Chiang Mai University, Chiang Mai, Thailand; 2 Innovation Center for Holistic Health, Nutraceuticals, and Cosmeceuticals, Faculty of Pharmacy, Chiang Mai University, Chiang Mai, Thailand; 3 School of Biomedical Sciences, Sri Balaji Vidyapeeth, Puducherry, India

**Keywords:** autism spectrum disorder, cognitive rigidity, gut microbiota, gut-brain axis, neuroimmune signalling, probiotics, synaptic plasticity

## Abstract

Autism spectrum disorder (ASD) is a heterogeneous neurodevelopmental condition characterised not only by social-communication difficulties but also by restricted interests, stereotyped behaviours, and marked cognitive rigidity. Over the past decade, converging lines of evidence have implicated gut dysbiosis, an imbalance in intestinal microbial composition and function, as a potentially important modulator of these behavioural phenotypes via the microbiota-gut-brain axis. In this narrative review, we integrate preclinical and clinical data to examine how specific microbial signatures, metabolic pathways, and immune and synaptic mechanisms may contribute to inflexible cognition in ASD. The manuscript outlines the organisation of the microbiota gut-brain axis in neurodevelopment and summarises reproducible microbial alterations reported in ASD cohorts. We then discuss how microbial metabolites, including short-chain fatty acids and tryptophan-derived neuroactive molecules, as well as immune mediators and neurotransmitter precursors, converge on pathways regulating excitatory-inhibitory balance, synaptic plasticity, and corticostriatal circuit function. Evidence from germ-free, genetic, and environmental rodent models provides causal support for microbiota-dependent modulation of repetitive and rigid behaviours, whilst clinical studies reveal associations between dysbiosis, metabolomic profiles, gastrointestinal symptoms, and ASD severity. Finally, we consider the translational landscape of microbiota-targeted interventions, probiotics, prebiotics, dietary strategies, and faecal microbiota transplantation and highlight key methodological and ethical challenges for moving toward precision microbiome-based therapies. Taken together, current data support gut dysbiosis as both a mechanistic contributor and a tractable therapeutic target for cognitive rigidity in ASD.

## Graphical abstract

**Figure:**
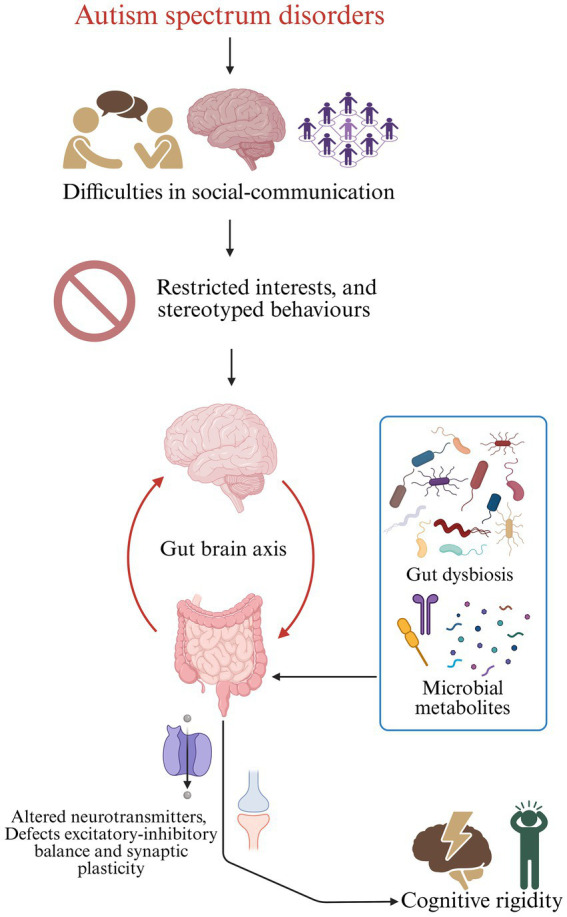
Conceptual model illustrating how gut dysbiosis contributes to cognitive rigidity in autism spectrum disorder (ASD). Individuals with ASD commonly exhibit difficulties in social communication, followed by restricted interests and stereotyped behaviours. These behavioural features intersect with alterations along the gut-brain axis. Gut dysbiosis, characterised by shifts in microbial composition and function, leads to abnormal production of microbial metabolites, including short-chain fatty acids and other neuroactive compounds. These metabolites influence the central nervous system through immune, metabolic, and neural pathways, resulting in altered neurotransmitter levels, disrupted excitatory-inhibitory balance, and impaired synaptic plasticity. Together, these processes contribute to increased cognitive rigidity, a core feature of ASD.

## 1 Introduction

The gut microbiota is a dense, metabolically active microbial community that interacts closely with host physiology. The microbiota-gut-brain axis links the intestine and the central nervous system (CNS) via neuronal, endocrine, immune, and metabolic signalling pathways (Mayer et al., 2022; Chakrabarti et al., 2022; Liu et al., 2022). Microbial communities regulate epithelial barrier integrity, shape mucosal and systemic immune responses, and generate bioactive metabolites that can reach the brain through the circulation or via neural routes such as the vagus nerve (Wang H. et al., 2023; Wang J. et al., 2023; Loh et al., 2024; Nakhal et al., 2024). Conversely, CNS activity, psychosocial stress, and neurodevelopmental perturbations alter gut motility, secretory patterns, and luminal habitat structure, reinforcing a bidirectional feedback loop between brain and gut (Mayer et al., 2022; Loh et al., 2024).

Autism spectrum disorder (ASD) is consistently associated with gastrointestinal (GI) comorbidities, most notably constipation, diarrhoea, and abdominal pain, and several recent clinical and systematic studies report that the burden of GI symptoms often parallels the severity of core social communication difficulties and repetitive behaviours (Lewandowska-Pietruszka et al., 2023; Wang H. et al., 2023; Wang J. et al., 2023; He et al., 2023; Fang et al., 2025). These converging data, together with experimental work in animal models, challenge a purely “brain-centric” view of ASD and instead support a systems-level framework in which peripheral physiology, including gut dysbiosis and mucosal immune activation, contributes to core cognitive and behavioural phenotypes (Lewandowska-Pietruszka et al., 2023; Fang et al., 2025). Germ-free and microbiota-depleted rodent studies have shown that the absence or disruption of commensal microbiota induces robust changes in anxiety-like behaviour, social interaction, stress responsivity, and cognitive flexibility, accompanied by alterations in synaptic protein expression, neurotrophin levels, and neurogenesis (Nagpal and Cryan, 2021; Wu et al., 2021; Delgado-Ocaña and Cuesta, 2024). Recolonisation with a conventional microbiota or defined microbial consortia can partially normalise these behavioural and molecular phenotypes, highlighting the importance of early-life microbial signals for the maturation of neural circuits that support flexibility and social behaviour (Nagpal and Cryan, 2021; Delgado-Ocaña and Cuesta, 2024; Al Noman et al., 2025).

**Figure 1:**
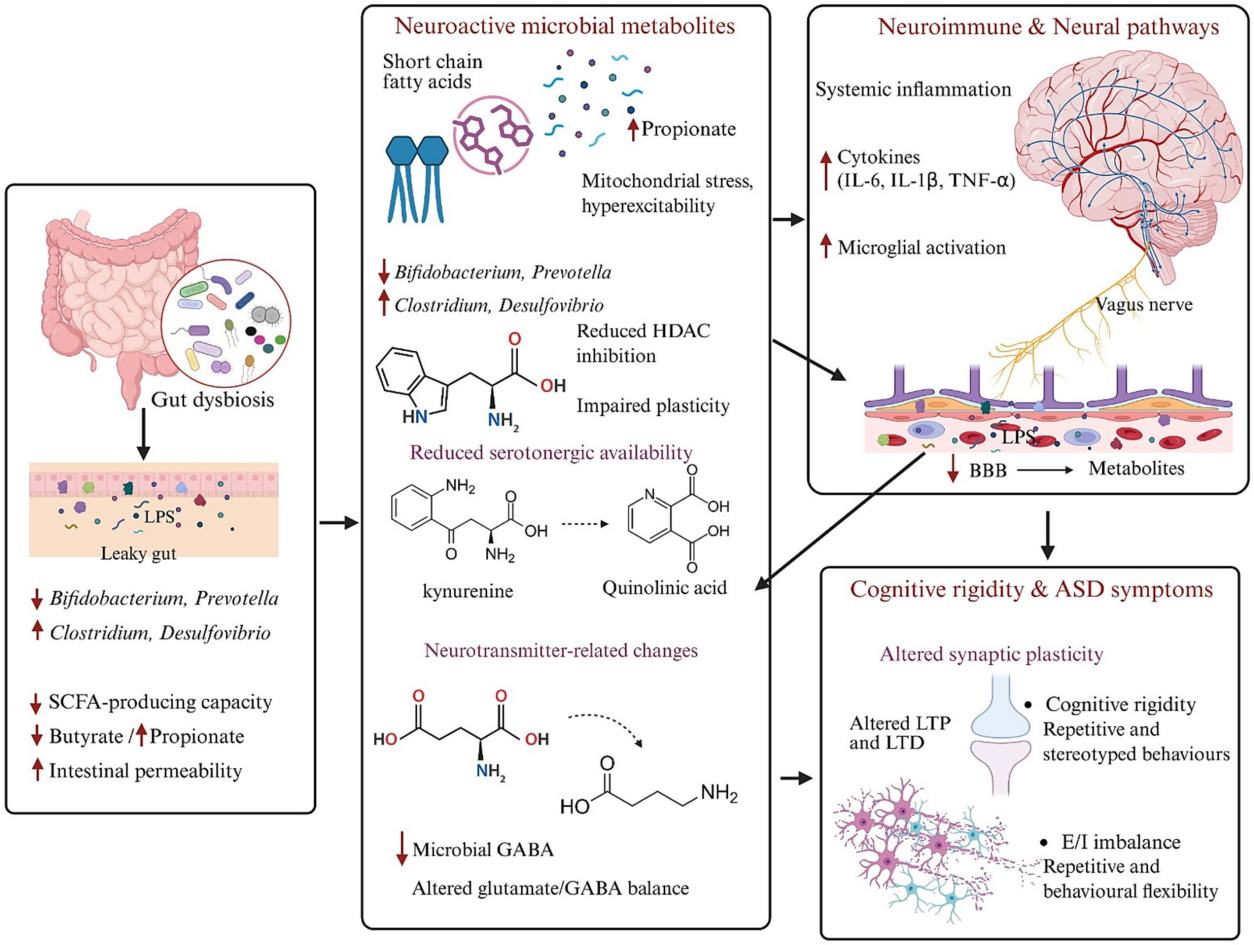
Mechanistic pathways linking gut dysbiosis to cognitive rigidity in autism spectrum disorder (ASD). Schematic representation showing how gut dysbiosis contributes to cognitive rigidity and core behavioural features of ASD via microbial, immune, and neural mechanisms. Alterations in gut microbial composition led to impaired gut barrier integrity and increased intestinal permeability. This disruption facilitates the translocation of microbial products into systemic circulation. Gut dysbiosis also alters the production of neuroactive microbial metabolites. Changes in short-chain fatty acid (SCFA) profiles are marked by increased propionate and reduced butyrate levels, which can promote mitochondrial stress, neuronal hyperexcitability, reduced histone deacetylase (HDAC) inhibition, and impaired synaptic plasticity. Simultaneously, dysregulated tryptophan metabolism with reduced serotonergic availability whilst alterations in neurotransmitter-related microbial pathways result in decreased microbial *γ*-aminobutyric acid (GABA) production and disturbed glutamate-GABA balance. These microbial and metabolic signals influence the brain through multiple routes, including systemic immune activation, blood–brain barrier (BBB) modulation, and vagus nerve signalling. Elevated circulating cytokines promote neuroimmune activation, particularly microglial activation, leading to aberrant synaptic pruning and sustained neuroinflammation. The convergence of immune, metabolic, and neural signalling disrupts synaptic plasticity, alters long-term potentiation (LTP) and long-term depression (LTD), and shifts the excitatory-inhibitory (E/I) balance within cortico-striatal and cortical circuits. Collectively, these processes impair neural flexibility and adaptive circuit function, culminating in increased cognitive rigidity, repetitive and stereotyped behaviours, and other core ASD-related symptoms.

Microbiota-derived metabolites, such as short-chain fatty acids, indole derivatives, tryptophan catabolites, and bile acid metabolites, modulate host gene expression, epigenetic programmes, and neurotransmitter synthesis, thereby influencing neuronal excitability and synaptic plasticity (Wang H. et al., 2023; Wang J. et al., 2023; Mansuy-Aubert and Ravussin, 2023; Zhang et al., 2023; Siddiqui et al., 2025). These same metabolites, together with microbial structural components, exert profound effects on systemic and CNS immunity, shaping microglial maturation, astrocyte reactivity, and activity-dependent synaptic pruning (Wang H. et al., 2023; Wang J. et al., 2023; Loh et al., 2024; Park et al., 2025). Vagal afferents provide a rapid neural conduit through which luminal and mucosal signals influence brainstem and limbic circuits; recent reviews emphasise that vagus-dependent signalling is required for the full behavioural impact of several microbiota-targeted interventions in preclinical models (Loh et al., 2024; Nakhal et al., 2024; Park et al., 2025). Within this framework, ASD related dysbiosis is plausibly positioned to disturb synaptic plasticity in prefrontal and cortico-striatal networks that underlie cognitive flexibility and behavioural adaptation, as suggested by emerging work linking microbiota-derived metabolites to circuit-level plasticity and network remodelling (Glinert et al., 2022; Al Noman et al., 2025; Fang et al., 2025) (Figure 1).

## 2 Gut dysbiosis and recurrent microbial profiles in autism spectrum disorder

Although considerable inter-individual variability exists, several traits of ASD-related dysbiosis have consistently emerged. Children with ASD often exhibit reduced microbial diversity and altered community structure compared to typically developing peers (Zou et al., 2021; Peralta-Marzal et al., 2024). At the compositional level, a recurring pattern includes the reduction of taxa typically regarded as beneficial and a proliferation of potentially pro-inflammatory or toxin-producing genera (including *Escherichia/Shigella*, *Sutterella*, *Eggerthella*, in addition to *Clostridium* and *Desulfovibrio*).

Reductions in the abundances of *Bifidobacterium* and *Prevotella* were the most notable findings. Recent analyses confirm depleted *Bifidobacterium* abundance in ASD cohorts, along with impaired barrier-supportive functions (Miao et al., 2024; Anaclerio et al., 2024). *Bifidobacteria* participate in carbohydrate metabolism, maintain epithelial integrity, and modulate immune signalling; thus, their reduction may heighten intestinal permeability and contribute to chronic low-grade inflammation. Likewise, the decline of *Prevotella* abundance, a key fermenter involved in short-chain fatty acid (SCFA) synthesis, has been consistently associated with reduced butyrate production and compromised mucosal health, with implications for synaptic and immunological pathways underpinning cognitive flexibility (Zhou et al., 2025).

In contrast, multiple recent studies report increased abundance of *Clostridium* and *Desulfovibrio* species in ASD microbiomes (Andreo-Martínez et al., 2022; Zhou et al., 2025). Certain *Clostridium* taxa produce neuroactive or neurotoxic metabolites capable of influencing host neurotransmitter systems, whereas *Desulfovibrio* is strongly linked to hydrogen sulphide generation and oxidative stress, with possible adverse effects on mitochondrial and neural function. Additional enrichment of Enterobacteriaceae-related taxa (*Escherichia/Shigella*) has been associated with elevated lipopolysaccharide burden and immune activation, further reinforcing a pro-inflammatory intestinal milieu. Although causality cannot be inferred from cross-sectional data, the observed shift from barrier-supportive, SCFA-producing organisms toward pro-inflammatory taxa aligns with mechanistic models of heightened neuroimmune activation and altered neurotransmission.

Beyond taxonomy, alterations in microbial metabolic capacity are increasingly recognised as essential. Metagenomic and metabolomic studies demonstrate ASD-related differences in vitamin biosynthesis pathways, sulphur metabolism, and SCFA production (Yap et al., 2021; Zhou et al., 2025). Notably, decreased butyrate-producing capacity and elevated propionate levels have been reported in recent ASD cohorts and translational animal models, suggesting that shifts in SCFA balance may influence neuronal excitability, plasticity, and immune tone (Andreo-Martínez et al., 2022). Certain taxa also show behaviour-linked associations; for example, enrichment of *Clostridium* species has been correlated with repetitive and stereotyped behaviours (Andreo-Martínez et al., 2022), whereas butyrate-producing and barrier-supportive taxa exhibit protective effects in preclinical systems (Miao et al., 2024). Despite persistent challenges, including variation in dietary patterns, sequencing platforms, and sample size, recent systematic reviews converge on the consensus that ASD is consistently associated with a dysbiotic microbiota marked by reduced diversity, loss of fermentative and mucosa-protective species, expansion of potentially pathogenic taxa, and altered metabolic function (Korteniemi et al., 2024). Collectively, these shifts may contribute to neuroimmune perturbation and cognitive rigidity.

## 3 Microbial metabolites and neuroactive molecules in cognitive rigidity

Microbial metabolites are pivotal signalling components of the gut-brain axis, maintaining long-term regulation of neuronal excitability, synaptic plasticity, and neuroimmune homeostasis, rather than merely serving as passive fermentation byproducts (Silva et al., 2020; O'Riordan et al., 2022; Petropoulos et al., 2025). Integrative metabolomic analyses of faecal, plasma, and urinary samples show that individuals with ASD exhibit consistent alterations in microbially derived metabolites linked to restricted interests, repetitive behaviours, and reduced cognitive flexibility (Needham et al., 2021; Lagod and Naser, 2023; Osredkar et al., 2025; Zheng et al., 2025).

Short-chain fatty acids (SCFAs), mainly acetate, propionate, and butyrate, are a crucial class of microbial metabolites that link dietary consumption to host cognitive function. SCFAs regulate epithelial barrier integrity, immune differentiation, microglial maturation, and the epigenetic modulation of gene expression in both the developing and mature brain through G-protein coupled receptor signalling and histone deacetylase inhibition (Silva et al., 2020; Cook and Prinz, 2022; Belén Sanz-Martos et al., 2022; Guo et al., 2023). In paediatric ASD populations, atypical SCFA profiles, marked by elevated propionate and variable acetate and butyrate levels, are frequently observed in conjunction with dysbiosis and gastrointestinal dysfunction (He et al., 2023; Lagod and Naser, 2023; Petropoulos et al., 2025). Experimental exposure to propionic acid in rodents supports a causal contribution by inducing ASD-relevant phenotypes, such as repetitive behaviours, social withdrawal, and impaired cognitive flexibility, in conjunction with mitochondrial dysfunction, oxidative stress, and abnormal glial activation, in a dose-dependent manner (Meeking et al., 2020; Hill et al., 2025). In contrast, butyrate and its derivatives provide neuroprotection and anti-inflammatory benefits, promote histone acetylation, and support hippocampal long-term potentiation and memory consolidation, highlighting the significance of balanced SCFA signalling for sustaining synaptic plasticity (Belén Sanz-Martos et al., 2022; Guo et al., 2023; Lan et al., 2024). Besides SCFAs, the microbial control of tryptophan metabolism provides an additional metabolic link between the gut microbiota and the neural circuits governing behavioural flexibility. The flux of tryptophan can be directed towards the synthesis of serotonin, kynurenine pathway metabolites, or indoles produced by the microbiota, each of which can distinctly influence neuroplasticity and neuroinflammatory tone (Li et al., 2022; Xue et al., 2023; Chen et al., 2024). Meta-analytic research demonstrates that ASD correlates with heightened kynurenine-pathway activity and altered tryptophan catabolite ratios, promoting the accumulation of neuroactive metabolites such as quinolinic acid, which affect glutamatergic transmission and microglial activation (Almulla et al., 2023). Preclinical studies indicate that elevated levels of kynurenine metabolites during prenatal or early life interfere with neurodevelopment and result in persistent social and repetitive behavioural impairments (Murakami et al., 2023). Considering the documented function of serotonergic signalling in limiting repeated behaviours and promoting behavioural flexibility, dysbiosis-induced alterations in tryptophan metabolism may directly exacerbate cognitive rigidity in ASD. The gut microbiome also influences the availability of classical neurotransmitters and their metabolic precursors. Various species of Lactobacillus and Bifidobacterium produce *γ*-aminobutyric acid (GABA), whilst other taxa modulate the metabolism of glutamate, dopamine, and catecholamines (Braga et al., 2024; Zhou et al., 2025). Integrated metabolomic and metagenomic analyses reveal disturbances in amino acid and vitamin metabolism in ASD, including alterations in glutamate–GABA pathways (Zhu et al., 2022; Zarimeidani et al., 2025). Systems-level profiling has indicated that microbial GABA imbalance constitutes a metabolic signature linked to impairments in social communication and restricted, repetitive behaviours, implying that the dysregulation of gut-derived neurotransmitters contributes to an excitatory–inhibitory imbalance within cortico-striatal and cortico-limbic circuits that promote cognitive flexibility (Kim and Shim, 2023; Wang et al., 2025).

Various classes of microbial metabolites, including SCFAs, aromatic phenolic compounds like p-cresol derivatives, and associated bioactive molecules, have been linked to oxidative stress, mitochondrial dysfunction, and compromised cellular bioenergetics (Dan et al., 2020; Liu et al., 2022; Hill et al., 2025). These cellular vulnerabilities are thoroughly described in ASD and are recognised to undermine synapse efficiency and plasticity. In accordance with these mechanistic insights, clinical metabolomic studies increasingly document convergent modifications in short-chain fatty acids, tryptophan catabolites, aromatic metabolites, and energy-related intermediates that correlate with the severity of restricted and repetitive behaviours and other fundamental symptom domains (Needham et al., 2021; Siracusano et al., 2023; Suárez-Jaramillo et al., 2025; Zheng et al., 2025). These findings identify microbial metabolites as active regulators of neuronal and cellular processes that contribute to cognitive rigidity in ASD, making them valuable candidates for future stratified and intervention-oriented studies.

## 4 Neuroimmune and neuroinflammatory pathways linking dysbiosis to cognition

The immune system plays a vital role in communication between the microbiota and the brain, and it appears to be constantly out of balance in individuals with ASD. Dysbiosis often links to increased intestinal permeability, which allows microbial components like LPS to enter the bloodstream (de Magistris et al., 2010; Bested et al., 2013). These substances activate pattern-recognition receptors such as toll-like receptors on immune cells outside the brain. This activation prompts immune cells to release pro-inflammatory cytokines like IL-6, TNF-*α*, and IL-1β (Fattorusso et al., 2019). Cytokines can cross the blood–brain barrier or communicate through endothelial and neuronal pathways, altering how the central nervous system functions. Numerous studies on ASD have shown that cytokine levels are often abnormal both in the body and in the brain. Typically, higher levels of IL-6, TNF-α, and chemokines like MCP-1 are observed (Hughes et al., 2024; Anastasescu et al., 2024). These mediators have dual roles in synaptic plasticity: at low levels, they support physiological pruning and long-term potentiation, but when chronically elevated, they impair plasticity and promote pathological pruning. Experimental alteration of IL-6 alone has been sufficient to disrupt foetal brain development and cause behavioural issues, such as difficulty with reversal learning and increased repetitive behaviours (Hu et al., 2025). This highlights the importance of cognitive rigidity. Microglia, the immune cells of the CNS, are highly sensitive to inflammatory signals and microbial products from external sources. Post-mortem analyses in ASD reveal increased microglial density and signs of chronic activation in the prefrontal and other cortical regions (Xiong et al., 2023).

Cytokine signalling caused by dysbiosis may keep microglia in a persistent pro-inflammatory state, leading to abnormal synaptic pruning and changes in network connectivity involved in flexibility and habit formation (Fan et al., 2023). Clinically, higher levels of inflammatory markers are linked to more severe restricted and repetitive behaviours. Some dietary interventions and probiotics that reduce inflammation can improve gastrointestinal symptoms and, in certain studies, slightly improve behavioural outcomes (Zhou et al., 2025). Overall, these findings support a model in which ASD-related dysbiosis causes ongoing, low-grade neuroinflammation that damages the synaptic structures essential for adaptive cognitive function.

## 5 Synaptic plasticity, excitatory-inhibitory balance, and microbial modulation

Synaptic plasticity enables learning, memory, and the ability to switch between different behavioural patterns. Extensive research shows that disrupting the balance of E/I signal and synaptic transmission plays a key role in the neurobiology of ASD, especially in prefrontal and corticostriatal circuits involved in cognitive flexibility, habit formation, and repetitive behaviours (Sylvester et al., 2025). Microbiota can influence these systems in several ways. As mentioned earlier, microbial production of GABA and the regulation of glutamate metabolism directly impact the E/I balance (Wang et al., 2025). A reduced presence of GABA-producing microbes, along with SCFA-related changes in receptor expression and synaptic proteins, may increase the susceptibility of networks to hyperexcitability and stereotyped firing patterns. Changes in gut microbial metabolism also affect monoaminergic systems. The serotonergic, dopaminergic, and noradrenergic pathways associated with ASD rely on signals from the periphery (Fang et al., 2025).

Germ-free animals provide substantial evidence that microbiota influence synapse structure. These mice show altered dendritic spine density, lower levels of synaptic proteins and BDNF in critical regions, and issues with long-term potentiation. Recolonisation can fix several defects, highlighting a key developmental window when microbial signals affect synaptic networks (Vuong et al., 2020). Human neuroimaging studies support these findings. Functional and structural MRI studies show that individuals with ASD have abnormal connections between cortical and basal ganglia networks, including circuits involved in cognitive flexibility. Initial research has started to connect these imaging findings with variations in gut microbial composition, though this area remains in early stages (Yap et al., 2021). These findings support the idea that dysbiosis negatively impacts synaptic plasticity and the E/I balance, restricting the neural capacity needed for adaptive thinking.

## 6 Animal models: from microbial perturbation to repetitive behaviour

Rodent models have been essential for investigating causal relationships amongst microbiota, cerebral function, and ASD-like symptoms. Germ-free mice demonstrate that the total lack of microbiota can lead to stereotypical grooming, modified stress responses, and deficits in reversal learning and social behaviour (Vuong et al., 2020). Colonisation with microbiota from neurotypical or ASD donors can differentially affect behaviour and neurochemistry, establishing a direct association between microbial communities and the emergence of ASD-like characteristics (Ruszkowski et al., 2025; Neyrinck et al., 2025). Genetic models, like BTBR T + tf/J mice and those with ASD-related mutations like Shank3, exhibit behavioural characteristics and atypical microbial profiles analogous to those observed in people (Lee et al., 2020; Alamoudi et al., 2022). Changing the gut microbiota in these animals with antibiotics, probiotics, prebiotics, or faecal transplants improves repetitive behaviours and social deficiencies, and it commonly also changes neuroinflammatory indicators and the E/I balance. Recent research on BTBR mice underscores the role of brain-resident T cells and particular probiotic strains, such as *Limosilactobacillus reuteri*, in reestablishing glutamate/GABA ratios and enhancing behaviours (Luo et al., 2023; Archer et al., 2025). Models of maternal immune activation and toxin exposure further substantiate the gut-immune-brain axis. Prenatal exposure to viral mimetics or bacterial components leads to children exhibiting ASD-like symptoms, gastrointestinal barrier dysfunction, dysbiosis, and neuroinflammation (Kim et al., 2022; Kim and Shim, 2023; Suprunowicz et al., 2024). In numerous models, prebiotic diets or probiotic strains can aid in re-establishing microbial equilibrium, mitigating inflammation, and partially normalising behaviours (Golbaghi et al., 2024; Yang et al., 2025). Overall, these preclinical investigations demonstrate that microbiota are active determinants affecting ASD-related behaviours, such as repetitive and inflexible activities, and that specific regulation of dysbiosis can enhance these characteristics.

## 7 Clinical correlates of microbiota, metabolites, and repetitive cognition

To translate findings from animal models into therapeutic practise, it is essential to meticulously assess microbiome-behaviour correlations within human ASD populations. Numerous case–control studies have validated that children with ASD display modified gut microbial composition in comparison to neurotypical counterparts, characterised by variations in diversity, taxonomic abundance, and community structure (Liu et al., 2019; Yap et al., 2021; Zou et al., 2021; Li et al., 2024; Zeng et al., 2025). Several studies have identified links between microbial characteristics and confined, repetitive habits or adaptive functioning. Metabolomic investigations of faecal, plasma, and urine samples indicate ASD-specific patterns in SCFAs, amino acid derivatives, and aromatic compounds, many of which are associated with microbial pathways (Yap et al., 2021; Needham et al., 2021). Increased propionate or related SCFAs, reduced butyrate levels, and modified tryptophan catabolites have been correlated with heightened stereotypy and stiffness (Meeking et al., 2020; Facchin et al., 2024).

Children exhibiting pronounced gastrointestinal symptoms frequently demonstrate more severe behavioural challenges and increased dysbiosis compared to individuals with ASD devoid of gastrointestinal complaints (Yap et al., 2021; Rose et al., 2018). This suggests that microbiota-induced intestinal dysfunction may exacerbate cognitive rigidity via immune and metabolic mechanisms. Interventional trials, however limited in scale, provide initial indications that microbiome modification can affect ASD symptoms. Microbiota transfer therapy (a form of standardised faecal microbiota transplantation (FMT) combined with antibiotics and bowel cleansing) in children with ASD resulted in lasting shifts toward a more “neurotypical-like” microbiota, sustained improvements in GI symptoms, and meaningful reductions in ASD behaviours, including repetitive actions, which persisted for up to 2 years in follow-up (Kang et al., 2019; Li et al., 2021; Xu et al., 2019; Li et al., 2024). Probiotic trials utilising *Lactobacillus* and *Bifidobacterium* strains have demonstrated moderate yet significant enhancements in social responsiveness and stereotypy in certain cohorts, frequently associated with partial normalisation of faecal microbial profiles (Liu et al., 2022; Wang H. et al., 2023; Wang J. et al., 2023; Li et al., 2024). Nonetheless, considerable obstacles persist. Diet, pharmaceutical use, and environmental exposures can obscure microbiome-behaviour associations (Xu et al., 2019; Yap et al., 2021). Sample sizes are generally limited, and methods differ significantly. It is challenging to establish causation in human populations, and the characteristics of responders and non-responders to microbiota-based therapy remain poorly defined. The convergence of compositional, metabolomic, and interventional data substantiates the hypothesis that gut microbiota and its metabolites significantly influence cognitive rigidity in ASD.

## 8 Therapeutic and future directions: toward precision microbiome interventions

The recognition that gut dysbiosis may contribute to the cause of ASD has opened a new therapeutic possibility (Mihailovich et al., 2024). Investigations are underway into strategies such as probiotics, prebiotics, dietary adjustments, and FMT to restore microbial equilibrium, ameliorate gastrointestinal symptoms, and perhaps affect fundamental behavioural concerns, including cognitive rigidity. Probiotic and “psychobiotic” formulations represent some of the most readily available adjuvant therapies. Strains like *Lactobacillus rhamnosus*, *Lactobacillus plantarum*, and *Bifidobacterium longum* have demonstrated beneficial effects on anxiety-related behaviours, social interactions, and stereotypic behaviours in animal models and initial human work (Mills et al., 2023). Early clinical trials report improvements in GI symptoms and modest but significant gains in social communication and repetitive behaviours (Phan et al., 2024; Zhang et al., 2022; Aghamohammad et al., 2023; Taha et al., 2025).

Prebiotics and specific dietary strategies seek to enhance favourable fermenters and elevate butyrate synthesis, hence promoting barrier integrity, immunological equilibrium, and neuronal plasticity (Lu et al., 2022; Phan et al., 2024). Despite ongoing controversy regarding gluten-free and casein-free diets, they may provide advantages for certain subgroups, highlighting the necessity for individualised strategies (Mavridou et al., 2025). FMT and associated microbiota transfer therapies constitute more extreme approaches, yielding promising yet preliminary outcomes in ASD (Zhang et al., 2023; Hu et al., 2023). Before the widespread use of these treatments, it is essential to standardise donor selection, dosage, and delivery techniques. Long-term safety monitoring will be necessary. Future trajectories in this domain are advancing towards precision microbiome-based therapy. The integration of metagenomics, metabolomics, host genetics, immunological profiling, and neuroimaging with machine learning could facilitate the identification of microbial signatures that characterise certain ASD subtypes and forecast treatment responses (Mihailovich et al., 2024; Taha et al., 2025; Mavridou et al., 2025).

Innovative approaches, including meticulously crafted microbial consortia, genetically modified probiotic strains that synthesise certain neuroactive compounds, and postbiotic formulations, demonstrate potential for more precise and safer therapies. Concurrently, ethical, regulatory, and practical concerns must be resolved, particularly when interventions pertain to infants and live microbial products with enduring ecological consequences (Taha et al., 2025). Microbiota-targeted therapies alone are unlikely to normalise cognitive stiffness; rather, they should be considered adjuncts that can augment the efficacy of behavioural and pharmacological treatments by reinstating biological substrates for plasticity. Gut dysbiosis is increasingly recognised not merely as a comorbidity but as a mechanistic aspect that contributes to cognitive rigidity in ASD (Zarimeidani et al., 2025). Meticulously structured, mechanism-oriented clinical trials, alongside stringent fundamental research, are essential for converting this information into dependable, individualised microbiome-based treatments to enhance outcomes in ASD.

## 9 Conclusion

Emerging evidence indicates that gut dysbiosis is not simply a secondary condition but a significant factor in the neurobiological mechanisms that contribute to cognitive rigidity in ASD. Convergent evidence from microbial, metabolomic, immunological, neurophysiological, and behavioural research indicates that modified microbial communities and their metabolites can compromise epithelial integrity, skew neuroimmune communication, and hinder synaptic development and E/I balance. Animal models offer causative evidence for these pathways, whilst initial clinical approaches suggest that microbiome modification can yield quantifiable, albeit inconsistent, behavioural enhancements. The amalgamation of metagenomics, metabolomics, and neuroimaging is set to enhance ASD subtyping and facilitate targeted microbiome therapies. However, methodological diversity and insufficient long-term data highlight the necessity for well-designed, mechanism-oriented experiments. The microbiota gut-brain axis presents a promising yet evolving framework for comprehending and potentially alleviating cognitive rigidity in ASD.

## 10 Heterogeneity and limitations in ASD microbiome

Human studies of the ASD gut microbiome are marked by considerable heterogeneity and occasional inconsistency, reflecting both methodological variability and biological stratification within ASD populations. Nevertheless, accumulating evidence supports the therapeutic potential of microbiota-targeted interventions, including antibiotics, prebiotics, probiotics, and faecal microbiota transplantation. Interpretation of these findings is limited by differences in study design, cohort composition, and analytical pipelines, highlighting the need for standardised frameworks to assess microbial composition, metabolite profiles, and inflammatory markers. Future research should prioritise longitudinal designs to evaluate the durability and developmental sensitivity of microbiome-based interventions. Identifying microbiota-derived metabolites that influence neurodevelopment will be essential for mechanistic insight and for advancing precision-oriented, individualised therapeutic strategies integrated with behavioural and caregiver-focused interventions.
